# RNAi in Pest Control: Critical Factors Affecting dsRNA Efficacy

**DOI:** 10.3390/insects16070737

**Published:** 2025-07-18

**Authors:** Maribel Mendoza-Alatorre, Brenda Julian-Chávez, Stephanie Solano-Ornelas, Tania Samanta Siqueiros-Cendón, Jorge Ariel Torres-Castillo, Sugey Ramona Sinagawa-García, María Jazmín Abraham-Juárez, Carmen Daniela González-Barriga, Quintín Rascón-Cruz, Luis Ignacio Siañez-Estrada, Edward Alexander Espinoza-Sánchez

**Affiliations:** 1 Laboratorio de Biotecnología I, Facultad de Ciencias Químicas, Universidad Autónoma de Chihuahua, Circuito Universitario S/N Nuevo Campus Universitario, Chihuahua C.P. 31125, Chihuahua, Mexico; p291285@uach.mx (M.M.-A.); p363961@uach.mx (B.J.-C.); tsiqueiros@uach.mx (T.S.S.-C.); qrascon@uach.mx (Q.R.-C.); lsianez@uach.mx (L.I.S.-E.); 2 Laboratorio de Nanotoxicología y Genómica Funcional, Centro de Investigación en Materiales Avanzados S.C., Miguel de Cervantes 120, Complejo Industrial Chihuahua, Chihuahua C.P. 31136, Chihuahua, Mexico; stephanie.solano@cimav.edu.mx; 3 Instituto de Ecología Aplicada, Universidad Autónoma de Tamaulipas, Ave. División del Golfo 356, Col. Libertad, Ciudad Victoria C.P. 87019, Tamaulipas, Mexico; joatorres@docentes.uat.edu.mx; 4 Laboratorio de Biotecnología, Facultad de Agronomía, Universidad Autónoma de Nuevo León, Francisco Villa S/N Col. Ex Hacienda El Canadá, General Escobedo C.P. 66050, Nuevo León, Mexico; sugey.sinagawagr@uanl.edu.mx; 5 Unidad de Genómica Avanzada, Centro de Investigación y de Estudios Avanzados del IPN, Libramiento Norte León Km 9.6, Irapuato C.P. 36821, Guanajuato, Mexico; jazmin.abraham@cinvestav.mx; 6 Laboratorio de Cultivo de Tejidos, División de Ingeniería y Ciencias, Tecnológico de Monterrey, Av. Heroico Colegio Militar 4700, Nombre de Dios, Chihuahua C.P. 31100, Chihuahua, Mexico; cgonzalezb@tec.mx

**Keywords:** RNA interference, pest management, dsRNA, design, delivery methods, biological barriers

## Abstract

In recent years, agricultural crops have increasingly been attacked by more destructive insect pests, forcing modern farming to depend mainly on chemical insecticides. Although valuable, their widespread and intensive misuse has raised serious concerns about environmental and public health impacts. RNAi has been proposed as a safer alternative due to its high specificity, adaptability, and low ecological footprint. So far, dsRNA has proven effective in controlling various pest species, either through topical application or via genetically modified plants. Despite advances, large-scale implementation of RNAi remains challenging due to technical and biological hurdles that contribute to inconsistent performance. Key aspects such as dsRNA design, delivery techniques, and cellular uptake mechanisms still require refinement. Additionally, ensuring environmental stability, addressing biosafety concerns, and developing cost-effective production methods are essential for its practical application. In this review, we explore recent advances in the design and implementation of dsRNA, as well as the strategies that could support the successful integration of RNAi technology into pest management programs.

## Simple Summary

In recent years, RNA interference (RNAi) has emerged as a promising technology for pest control by silencing essential genes, with high specificity and low environmental impact, which makes it a viable alternative to chemical insecticides. However, its effectiveness is influenced by several factors, including the length and structure of double-stranded RNA (dsRNA), the target insect species, microbiome interactions, cellular uptake, and environmental conditions. A better understanding of these aspects could help establish guidelines that improve RNAi effectiveness; therefore, in this review, we explore recent advances in the use of RNAi for insect pest control and discuss key issues that must be addressed for its large-scale practical implementation.

## 1. Introduction

Ensuring global food security is one of the most critical challenges of the 21st century; although efforts have been made to increase agricultural productivity, factors such as climate change, soil degradation, and resource scarcity continue to hinder our ability to meet growing food demands [1].

Insect pests represent another major constraint, responsible for up to 38% of crop yield losses, with associated global economic damage estimated at USD 470 billion annually [2]. To mitigate the impact of pests, several management strategies have been implemented, including cultural practices and biological control; however, the primary control method has been the application of chemical insecticides [3]. Despite contributing to food production, the indiscriminate use of insecticides has led to environmental contamination and significant risks to human health [4,5,6,7], with approximately 150,000 deaths annually due to pesticide poisoning [8]. Moreover, insecticides have unintended effects, including impacts on non-target organisms such as pollinators and natural enemies [9,10,11], as well as the development of resistance in pest populations [12,13,14]. As of 2025, 19,500 cases of resistance have been reported across 634 pest species [15].

Due to the problems associated with insecticides, new strategies have been reported to enhance pest control. Among them, RNAi has gained significant attention due to its high specificity, reduced likelihood of non-target effects, and low environmental impact [16].

**Figure 1:**
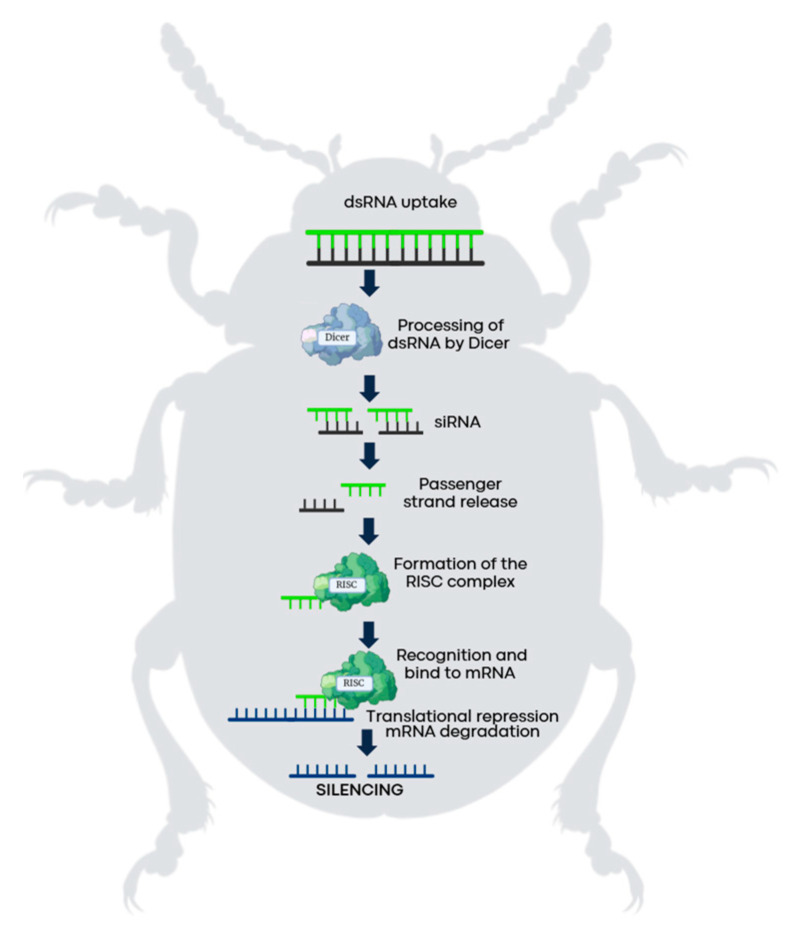
RNA-mediated silencing mechanism in pests. The process begins when the pests ingest dsRNA molecules, which enter the cell and are recognized by the Dicer protein. Dicer processes the dsRNA into small interfering RNAs that are loaded into the RISC complex, which mediates the sequence-specific degradation of complementary mRNA.

RNAi is a regulatory mechanism of gene expression that acts through the selective degradation of specific messenger RNAs (mRNA) [17,18], thereby preventing the production of encoded proteins [19]. The process is triggered by the presence of dsRNA molecules in the cell, which are recognized by a ribonuclease III-type enzyme, Dicer-2. This enzyme cleaves the dsRNA into small fragments of approximately 21–25 nucleotides in length, typically bearing 2-nucleotide 3′ overhangs on each strand. These fragments are known as small interfering RNAs (siRNAs) [20,21,22,23]. The resulting siRNAs are subsequently incorporated into the RNA-induced silencing complex (RISC), where the Argonaute-2 protein facilitates the sequence-specific cleavage of complementary mRNA targets [20,24] (Figure 1).

Due to its capacity for silencing specific genes, RNAi has been employed in insect pests to disrupt essential physiological processes such as development, metabolism, and reproduction [25,26,27]. This strategy has been applied in a wide range of species, including *Frankliniella occidentalis* (Thysanoptera: Thripidae), *Chilo partellus* (Lepidoptera: Crambidae), *Drosophila suzukii* (Diptera: Drosophilidae), *Leptinotarsa decemlineata* (Coleoptera: Chrysomelidae), *Spodoptera frugiperda* (Lepidoptera: Noctuidae), *Locusta migratoria* (Orthoptera: Acrididae), *Tetranychus urticae* (Trombidiformes: Tetranychidae), *Diaphorina citri* (Hemiptera: Liviidae), *Myzus persicae* (Hemiptera: Aphididae), *Bemisia tabaci* (Hemiptera: Aleyrodidae), *Aphis gossypii* (Hemiptera: Aphididae), *Helicoverpa armigera* (Lepidoptera: Noctuidae), *Tuta absoluta* (Lepidoptera: Gelechiidae), *Tribolium castaneum* (Coleoptera: Tenebrionidae), and *Panonychus citri* (Trombidiformes: Tetranychidae) [16,17,26,28,29,30,31,32,33,34,35,36,37,38,39]. The application of RNAi in these species has resulted in significant knockdown and phenotypic effects, demonstrating its potential in pest management.

**Figure 2:**
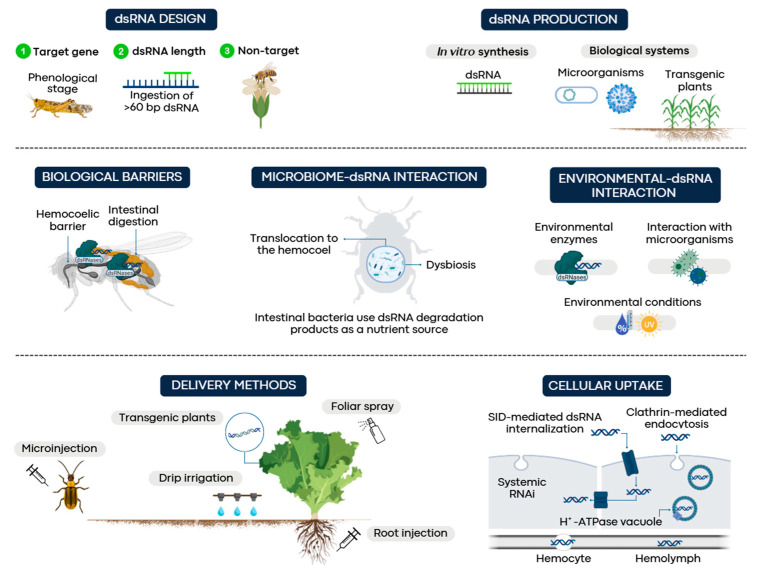
Factors affecting the efficiency of RNAi-mediated gene silencing in insects.

Although RNAi has shown promising results in pest management, insect physiological responses remain variable due to multiple biological and environmental aspects that limit its large-scale application (Figure 2). This review explores the key factors influencing RNAi effectiveness, including dsRNA design, production systems, delivery and uptake mechanisms, intracellular transport, biological barriers in insects, environmental and microbiome interactions, and the risks of resistance development. It highlights current challenges and opportunities to enhance RNAi-based pest control strategies.

## 2. Design of Double-Stranded RNA for Gene Silencing

dsRNAs trigger the RNAi mechanism, but their effectiveness in target organisms largely depends on dsRNA length and the identity of the target mRNA. When designing dsRNA molecules for gene silencing in pest management, it is crucial to consider these factors to maximize gene silencing efficiency.

### 2.1. dsRNA Length

Although gene silencing is ultimately mediated by 21–25 nt siRNAs, the length and composition of the dsRNA molecule from which they are derived significantly influence the effectiveness and specificity of pest control. For example, short dsRNAs (<27 nt) often exhibit limited knockdown efficiency compared to longer dsRNA molecules (>60 nt) [40,41,42]. This reduced efficiency is likely due to two primary factors: first, reduced uptake across the insect midgut epithelium, as reported in *Diabrotica virgifera virgifera* (Coleoptera: Chrysomelidae) for the *Snf7* and *v-ATPase C* genes [40,43]; second, fewer siRNAs generated after Dicer processing, which decreases the likelihood of effective target mRNA degradation [44,45].

Because of this, many studies have focused on the use of long dsRNAs. A positive correlation between dsRNA length and silencing efficiency has been observed, for example, in *T. castaneum*; longer dsRNAs were found to be more effective in silencing *CHS2* and *NAG2* genes [41]. However, no consensus exists regarding the optimal dsRNA length threshold required for effective gene silencing. To date, a broad range of dsRNA lengths has been successfully employed to silence distinct target genes in different species. In *L. decemlineata*, gene silencing has been achieved using dsRNA of varying lengths: *Sec23* (1506 bp), *ATPase E* (469 bp), *ATPase B* (530 bp), *COPβ* (228 bp), *β-actin* (298 bp), *Prohibitin-1* (350 bp), *Shd* (438 bp), *SAHase* (521 bp), *Ran* (361 bp), *NAT1* (357 bp), *HR3* (141 bp), *ACE1* (670 bp), and *EcR* (445 bp) [46,47,48,49,50,51,52,53,54]. Whereas in *D. virgifera virgifera*, dsRNA of 240 bp and 184 bp were used to silence *Snf7* and *v-ATPase C* genes, respectively [40,43]. Similarly, a 220 bp dsRNA successfully silenced the *β-actin* gene in *B. tabaci* [55], and 189 bp was effective in *H. armigera* [56].

These studies clearly indicate that dsRNA is a key determinant of RNAi success; however, its influence is modulated by other elements such as target gene identity, mRNA region, and sequence context [54,57]. Silencing efficiency has been shown to vary even when dsRNAs of equal length target different positions of the mRNA [40], suggesting that structural accessibility, GC content, and the functional relevance of the targeted region should also be considered [54]. Consequently, determining the optimal dsRNA length is not merely a matter of size but must be empirically optimized based on the target gene and insect species, ideally incorporating bioinformatic predictions of secondary structure and sequence conservation.

### 2.2. Target Sequence

Selecting a target gene is one of the most critical factors to be considered in achieving effective silencing through RNAi. More than 90 genes have been successfully silenced across over 30 insect species, mainly involved in essential physiological processes such as homeostasis, development, reproduction, metabolism, detoxification, and cellular integrity [26,29,36].

However, successful silencing does not solely depend on the biological function of the target gene but also on intrinsic molecular characteristics that directly affect the efficiency of the process; therefore, a deep understanding of insect biology as well as insight into the specific molecular and physiological roles of the genes involved is essential. In this context, several genes have been cataloged as effective targets due to their consistent phenotypic effects across diverse insect species [12,28,29,58,59] (Table 1).

One of the most extensively studied targets is the *V-ATPase* gene, which is involved in maintaining ion gradients and pH homeostasis. Silencing of various *V-ATPase* subunits has been shown to disrupt essential physiological processes, resulting in high mortality rates [29,31,38,60,61]. Another frequently targeted gene is chitin synthase (*CHS*), which is essential for cuticle formation and the integrity of the peritrophic matrix; its inhibition results in developmental defects, molting abnormalities, and delayed growth [36,59,74]. Acetylcholinesterase (*ACE*) has also been silenced due to its role in terminating synaptic transmission, resulting in acetylcholine accumulation, leading to severe neurotoxic effects, loss of neuromuscular function, and ultimately, death [29,33,35,54].

Nevertheless, silencing essential genes does not guarantee an effective RNAi response. Factors such as mRNA stability, large transcripts, and the accessibility of the region on the target gene can resist degradation, attenuating the silencing response [75,76,77]. Therefore, it is recommended to avoid non-coding regions or sequences too close to the start codon, as these could hinder the binding of the silencing machinery (RISC) due to the presence of secondary structures or regulatory proteins. However, this is a point that is widely discussed [16,40,57]. The coding sequence (CDS) is a more suitable target, particularly segments with minimal secondary structure and moderate thermodynamic stability [78]. Studies such as Schubert et al. [79] demonstrated that regions with high folding free energy (ΔG) are more accessible to DICER processing and RISC loading. Significantly, the ΔG is related to GC content because excessively high GC content may lead to the formation of highly stable hairpins or internal loops that hinder processing [80,81]. Therefore, moderate GC content (approximately 50%) can provide enough stability to extend the dsRNA lifespan in the cellular environment without affecting its processing [78].

Another important factor is the turnover rate of the target proteins. Proteins with long half-lives may persist even after mRNA levels are significantly reduced, delaying or diminishing observable phenotypic effects [82]. Gene expression levels and functional redundancy also influence RNAi outcomes: highly expressed genes may require higher doses of dsRNA [83,84]. In contrast, compensatory genes may mitigate the effects of silencing a single target gene [85]. Additionally, compensatory feedback mechanisms may arise in response to gene knockdown. For instance, Willow et al. [67] reported that RNAi-mediated silencing of the *αCOP* gene in *Brassicogethes aeneus* (Coleoptera: Nitidulidae) led to post-treatment overexpression of the same gene. This mechanism may undermine the effectiveness of RNAi applied to insects. Therefore, one strategy is to select genes that do not have associated compensatory genes or redundant pathways.

Although selecting an appropriate target sequence is critical, the susceptibility of the target species must be considered. While the RNAi machinery is conserved across many insect taxa, species-level differences significantly influence RNAi efficiency. For example, insects in the order Coleoptera exhibit a higher susceptibility to RNAi than Lepidoptera [86,87,88]. This differential RNAi sensitivity may be attributed to lineage-specific factors, gene copy number, or variations in gut enzymatic activity and biochemical environments, which reflect each species’ dietary and ecological adaptations [18]. Moreover, the expression of core RNAi components fluctuates across life stages [24,89]. For example, differential mortality was observed in larvae and adults of *Musca domestica* (Diptera: Muscidae) and *Delia radicum* (Diptera: Anthomyiidae) following RNAi treatment [23], highlighting that the development stage and the target tissue are factors to be considered [84,90].

Although it is not possible to predict the physiological outcome of silencing a particular gene, its selection remains a crucial step. Recently, transcriptomic analyses have emerged as a powerful tool for identifying RNAi-sensitive genes, as shown in *Phenacoccus solenopsis* (Hemiptera: Pseudococcidae) and *Amrasca biguttula* (Hemiptera: Cicadellidae), where novel genes involved in fertility, egg nutrition, neurotransmission, and insecticide detoxification were identified [89,91]. Despite this progress, in-depth functional studies are still required to understand gene–gene interactions and their combined influences on RNAi efficacy. Nonetheless, in vivo empirical validation in different ecological contexts remains essential if we are to confirm the feasibility of candidate target genes.

### 2.3. Non-Target Effects

While dsRNA has been proposed as a highly targeted pest control strategy, its large-scale use raises some concerns. Once released, dsRNA can interact with non-target organisms, such as pollinators, phylogenetically related species, or even mammals, causing unintended gene silencing. These interactions raise important safety considerations for the environmental use of RNA-based pesticides [92].

Several studies have attempted to address these concerns, offering some reassurance. For example, DP23211 and MON 87411 maize, which produce dsRNA targeting *D. virgifera virgifera*, have demonstrated safety in birds, pollinators, and aquatic species [93,94,95,96], even when applied at concentrations exceeding the maximum expected environmental levels. However, such findings cannot be generalized to other dsRNA constructs without similarly robust evidence.

There are also studies that show non-target effects. For instance, Chen et al. [97] showed that the dsRNA designed to target the *rpl19* gene from *Bactrocera dorsalis* (Diptera: Tephritidae) caused knockdown in the *rpl19* genes of *Bactrocera minax* (Diptera: Tephritidae) and *Diachasmimorpha longicaudata* (Hymenoptera: Braconidae). Likewise, Powell et al. [23] reported that dsRNA targeting the *Diap1* gene from *D. radicum* caused non-target effects on *M. domestica*, and, surprisingly, non-specific gene downregulation was reported by Jarosch and Moritz [98] in *Apis mellifera* (Hymenoptera: Apidae).

On the other hand, it has been proposed that plant-derived dsRNAs do not pose a hazard to mammals such as mice or humans—even at oral doses millions to billions of times higher than anticipated human exposures—due to the numerous biological barriers that limit uptake and potential for activity [99,100]. However, caution is warranted before concluding that plant-produced dsRNA is entirely harmless. Several factors, such as off-target interactions, may still raise concerns. siRNAs can interact with transcripts that share only partial sequence similarity [101]. In some cases, long dsRNA molecules may unintentionally suppress genes [102]. It has been suggested that RNA molecules, including dsRNAs from diet or the environment, could be absorbed and affect human biology [103,104,105]. More research is needed to confirm this.

Beyond gene silencing, in mammals, dsRNA can trigger immune responses. This includes the release of inflammatory molecules like TNF and interferons [106,107]. Receptors like TLR3, RIG-I, and MDA5 detect dsRNA and start immune signaling. This can lead to immune activation or block protein synthesis [108,109]. Moreover, high levels of dsRNA may saturate the RNAi machinery, interfering with normal gene regulation [110,111]. In addition to immune signaling, dsRNA can elicit broader immunogenic effects, such as cellular stress and apoptosis [112,113]. Therefore, case-by-case comprehensive risk evaluations are essential [114].

Currently, there is a lack of consensus on how and why certain organisms are susceptible or unresponsive to dsRNA. Although non-target effects are typically attributed to sequence similarity, recent findings suggest that this criterion alone may be insufficient. While gene silencing in non-target organisms often requires a continuous sequence identity of 26–32 bp [44,115], some studies have reported silencing effects with as few as 15 bp, or even 11 bp, in phylogenetically related non-target species [23,44,101]. In contrast, Castellanos et al. [116] reported that 17–21 bp of identity between *Euschistus heros* (Hemiptera: Pentatomidae) and egg parasitoid *Telenomus podisi* (Hymenoptera: Platygastridae) was insufficient to induce gene expression changes. These findings indicate that non-target effects are influenced by factors beyond sequence similarity, including mRNA accessibility, tissue expression patterns, delivery method, and even the predominant length of the siRNAs resulting from processing exogenous dsRNA, which is dependent on the species [117].

Furthermore, in *A. mellifera*, Nunes et al. [118] reported that a non-specific dsRNA-GFP altered the expression of approximately 10% of the transcriptome. While some changes were linked to partial sequence complementarity, many appeared to result from broader physiological responses, including RNA processing and transport, hormone metabolism, immunity, and responses to external stimuli and stress. These findings suggest that factors such as dsRNA structure may also contribute to unintended effects.

To date, there is no unified and predictive model for non-target risk assessment, which reveals a critical knowledge gap, and the current paradigm, based solely on bp identity, oversimplifies the biological complexity of RNAi responses across species. A more robust strategy is needed that integrates ecological interactions and transcriptomic profiles of non-target organisms likely to encounter dsRNA [44,119]. Another concern is the potential for dsRNA to move through the trophic chain, affecting predators or other non-target organisms exposed to the dsRNA [115,120].

While many studies report no observable non-target effects [121], these results should be interpreted with caution, as most biosafety assessments are based on a limited number of taxa and experimental conditions. Moreover, immune responses triggered by dsRNA are sequence-independent [113], meaning that even the careful selection of target sequences cannot fully mitigate non-target effects. Under these conditions, the true ecological risk may be underestimated.

A better understanding of how dsRNAs are processed and loaded into RISCs and what degree of mismatch they can tolerate is necessary to improve non-target predictions. However, current computational models remain insufficient [122]. In the absence of clear guidelines about dsRNA design, avoiding highly conserved regions in homologous genes is advisable to minimize the risk of silencing genes in non-target organisms [123].

The environmental impact of dsRNA on organisms surrounding the application site is also a concern, particularly in soils and in aquatic ecosystems that support diverse ecological functions and services [124,125]. Although rapid degradation of dsRNA has been reported [126], this alone does not guarantee ecological safety. There is a window of time, ranging from hours to days, during which dsRNA remains biologically active in the environment. This persistence varies depending on environmental conditions and dsRNA-specific characteristics, complicating risk assessments. Therefore, more holistic evaluations are needed, ones that include sequence identity analysis, ecological and transcriptomic data, and incorporate environmental variability and diverse dsRNA formulations to ensure the safe and effective implementation of RNAi-based pest control.

## 3. dsRNA Production Systems

A robust RNAi response depends fundamentally on the continuous supply of dsRNA to trigger gene silencing [127]. However, despite technical advances, the scalable, safe, and cost-efficient production of dsRNA remains one of the main bottlenecks for large-scale agricultural implementation.

One commonly used approach to produce dsRNA is in vitro synthesis, typically catalyzed by T7 RNA polymerase, using DNA templates that incorporate T7 promoter sequences, either with convergent promoters to produce dsRNA or inverted repeats separated by a spacer to produce a hairpin RNA (hpRNA) [128,129]. Although each strategy has limitations, such as the additional processing steps to ensure strand pairing to form dsRNA and the heterogeneous folding and secondary structures associated with hpRNA, in vitro synthesis can produce high-purity dsRNA, making it particularly suitable for controlled laboratory experiments and gene function studies. It has been successfully applied to silence target genes in insects such as *P. citri*, *H. armigera*, *F. occidentalis*, *L. decemlineata*, and *S. frugiperda* [30,31,32,83]. However, the high production costs and the environmental instability of naked dsRNA molecules [130,131] limit the scalability of this approach.

To reduce the economic impact of dsRNA production, microbial systems have been employed due to their high scalability, enabling a significant reduction in production costs (approximately USD 4 per gram) [66,131]. Among these, the *Escherichia coli* HT115 (DE3) strain, which lacks RNase III activity, has been extensively used [25,29], offering the flexibility to extract dsRNA or apply live or inactivated *E. coli* cells directly to the target pest. However, both the bacterial strain and fermentation conditions are critical factors influencing dsRNA yield [128,130,132]. Other bacteria, including *Bacillus subtilis*, *Bacillus thuringiensis*, and *Corynebacterium glutamicum*, have also shown potential as dsRNA production platforms [133,134,135]. Yeasts such as *Saccharomyces cerevisiae* have been explored due to their lack of Dicer-2 and Argonaute-2 genes, which enable the intracellular accumulation of long RNA molecules [92,136]. Because some are classified as GRAS yeasts (generally recognized as safe yeasts), they offer additional regulatory advantages for agricultural applications [128,130]. A related approach involves the use of viroid-based RNA scaffolds combined with self-splicing introns to enhance the yield and scalability of dsRNA production [22]. Additionally, components of bacteriophages have been incorporated into production systems. For example, non-pathogenic strains of *Pseudomonas syringae* have been engineered to constitutively express a DNA-dependent T7 RNA polymerase (T7 DdRp) from bacteriophage Φ6, a single-stranded RNA (ssRNA) template, and a Φ6 capsid protein along with the T7 RNA-dependent RNA polymerase (T7 RdRp) [128]. The transcribed ssRNA is encapsulated within the phage capsid, which protects the dsRNA from degradation [129,132,137]. This system has been primarily applied to antiviral strategies.

An emerging approach is symbiont-mediated RNAi (SMR), which exploits the natural association between symbiotic microorganisms and their insect hosts [138]. For this, the symbionts are genetically engineered to continuously express dsRNA, serving as both producers and delivery vehicles [139]. This was validated using engineered *Serratia symbiotica* to express dsRNA targeting the *C002* and *EcR* genes in the gut of the pea aphid (*Acyrthosiphon pisum*, Hemiptera: Aphididae) [140]. Other examples include *Rhodococcus rhodnii* and the bacterial symbiont BFo2 of *F. occidentalis*, which have also been modified to produce dsRNA, resulting in significant larval mortality [141]. However, despite the promising potential, inherent risks such as the uncontrolled dispersal of genetically modified symbionts and horizontal gene transfer pose significant environmental concerns.

Although dsRNA production systems have been improving, the development of plants that express continuous and stable dsRNAs is a key strategy for the scalability of RNAi technology. Routinely, the dsRNA cassettes are integrated into the nuclear genome, and the cell machinery is responsible for dsRNA production. To date, this strategy has shown promising results. For instance, the expression of dsRNA in tobacco plants targeting *Spodoptera litura* (Lepidoptera: Noctuidae), *C. partellus*, and *Plutella xylostella* (Lepidoptera: Plutellidae), resulted in lethal phenotypes and larval weight reduction [142]. Other crops such as pigeonpea (*Cajanus cajan*), tomato (*Solanum lycopersicum*), maize (*Zea mays*), and potato (*Solanum tuberosum*) have also been developed [58,143,144,145]. A fundamental limitation of nuclear expression is its inherent conflict with the cell’s own RNAi machinery, which processes dsRNA and reduces the amount of unprocessed dsRNA or hpRNA available for pest ingestion [16,146]. This complicates the long-term viability and efficiency of nuclear-expressed dsRNA in the field, especially under variable environmental conditions.

Due to limitations associated with nuclear expression of dsRNA, chloroplasts have been explored as an alternative for high-level dsRNA production. Currently, crops such as tobacco, tomato, and potato have been provided with protection against *L. decemlineata*, *H. armigera*, *Henosepilachna vigintioctopunctata* (Coleoptera: Coccinellidae), *M. persicae*, *F. occidentalis*, *Tetranychus evansi* (Trombidiformes: Tetranychidae), *Tetranychus truncatus* (Trombidiformes: Tetranychidae), and *Tetranychus cinnabarinus* (Trombidiformes: Tetranychidae) [16,56,65,121,147]. The expression of dsRNAs in the chloroplast is a relatively recent area of interest, and certain knowledge gaps remain to be addressed. For instance, the maximum size of expression cassettes that can be stably integrated into the chloroplast genome and the optimal length of dsRNA molecules produced in this organelle require further investigation. Careful consideration of these factors is essential: although dsRNAs longer than 60 nt are generally effective for gene silencing, there appears to be a negative correlation between dsRNA length and its accumulation in chloroplasts [42,148]. Another important challenge is that chloroplast transformation is not established in all crops, mainly monocots, which limits its implementation in agriculture. However, despite these challenges, the dsRNA expression in plastid compartments offers advantages such as the absence of RNAi-processing machinery within plastids and the bioencapsulation of dsRNA that protects it from degradation [127,149]. Moreover, dsRNA expression is mainly restricted to photosynthetic tissues, which adds a safety advantage for the consumption of other plant parts, such as tubers, roots, and seeds.

## 4. Delivery, Uptake, and dsRNA Transport

### 4.1. Delivery Methods

For RNAi to induce significant physiological effects in pests, each dsRNA molecule must exert the greatest possible impact on the target organism. This largely depends on the delivery method used. Although highly efficient methods such as immersion and microinjection are widely employed in laboratory settings, they are labor-intensive and impractical for field application [143,150,151]. Therefore, scalable strategies for exogenous dsRNA delivery have been developed and tested under realistic agricultural conditions.

One of the most prominent field-compatible approaches is spray-induced gene silencing (SIGS), in which dsRNA is applied to plant surfaces. The effectiveness of this method has been demonstrated in several studies. In *Hordeum vulgare*, for example, dsRNA sprays reduced gene expression by 60% in *Sitobion avenae*, demonstrating phloem-mediated uptake and systemic transport [152]. Similar results were reported in *Fraxinus pennsylvanica*, where foliar-applied dsRNA targeting *Agrilus planipennis* (Coleoptera: Buprestidae) triggered RNAi effects lasting up to 21 days [153]. Other successful applications include the control of *B. aeneus* in *Brassica napus* and the protection of stored *Oryza sativa* grains from *Rhyzopertha dominica* (Coleoptera: Bostrichidae) [154,155]. In 2019, Bayer submitted a sprayable RNAi biopesticide developed with BioDirect™ technology to the U.S. Environmental Protection Agency (EPA) for registration to control the varroa mite. More recently, Calantha^TM^ (Ledprona) has been approved by the EPA [156,157]. Additionally, other sprayable RNAi products are still under development, including those targeting the canola flea beetle, diamondback moth, and Colorado potato beetle [158,159,160].

While foliar application is effective, other methods may offer advantages in specific contexts. Root drenching, for instance, enables the uptake of dsRNA through the root system and subsequent translocation throughout the plant. This approach was validated in tomato, where root-applied dsRNA induced silencing of *AChE*, *nAChRs*, and *RyR*, causing mortality in *T. absoluta* [62]. Systemic movement and persistence have also been documented in *F. pennsylvanica*, where dsRNA remained detectable for up to 30 days [161]. In addition, trunk injection, which facilitates dsRNA distribution via the vascular system, has also been tested in *Malus pumila*, *Citrus macrophylla*, and *Vitis vinifera* [162,163,164]. This method has proven effective with detectable dsRNA up to seven weeks post-injection.

Although SIGS is an attractive approach, environmental factors and microbial degradation can reduce dsRNA stability under field conditions, making it difficult to achieve consistent efficacy. Meanwhile, root drenching and trunk injection may be affected by limited penetration due to cell walls [132].

To address these challenges, various strategies have recently been developed to enhance dsRNA delivery. For example, fusion proteins based on lectins have been used as RNAi carriers in lepidopteran pests [165]. Nanoparticle-based systems have also emerged as promising delivery platforms, offering protection, sustained release, and improved cellular uptake. These include poly-L-lysine (PLL) combined with the polyphenol epigallocatechin gallate, amine-functionalized mesoporous silica nanoparticles, hollow mesoporous silica, cationic dendrimers, chitosan, and biodegradable chitosan-based hydrogels [26,59,166,167,168,169,170]. Similarly, layered double hydroxide clay nanosheets—commercially known as BioClay^TM^—have shown the ability to stabilize dsRNA on leaf surfaces, enabling gradual release and sustained gene silencing for up to 30 days, even in newly emerged, unsprayed leaves [171,172].

Other innovative tools include virus-like particles derived from *Drosophila* X virus, used to encapsulate and deliver dsRNA into insect cells, as well as cell-penetrating peptides (CPPs), which enhance dsRNA uptake in plant tissues [38,173].

A key factor that must be considered is dsRNA dosage. There is no universal dose as it varies depending on factors such as the target gene, insect species, and developmental stage [174,175]. For instance, field applications may require relatively high quantities from 2–10 g ha^−1^ of in vitro-synthesized dsRNA to achieve effective gene silencing [175]. In contrast, laboratory-based methods typically involve low doses. To silence *ACE1*, *SERCA*, and *CPR* genes in *L. decemlineata*, 4–8 ng μL^−1^ of dsRNA were used [54,176]. Similarly, for *Psylliodes chrysocephala* (Coleoptera: Chrysomelidae), 25–200 ng cm^−2^ of dsRNA was sufficient to silence *Sec23* and *VatpG* [177]. Additionally, the route of administration also has an influence. In the oriental stink bug (*Plautia stali*, Hemiptera: Pentatomidae), 30 ng of dsRNA was sufficient for effective silencing using microinjection, whereas oral delivery required up to 5 μg μL^−1^ [178]. A comparable trend was observed in Asian citrus psyllid (*D. citri*) when 200 ng μL^−1^ of orally supplied dsRNA was needed to achieve effective silencing [174].

Finally, transgenic plants can be used to deliver dsRNA through a process known as host-induced gene silencing (HIGS). This method offers a stable and targeted alternative for pest control. As mentioned earlier in the section about dsRNA production, HIGS enables continuous synthesis of dsRNA in plants. This reduces the need for repeated applications and helps provide longer-lasting protection against pests.

### 4.2. Cellular Uptake

Effective RNAi-mediated gene silencing requires the successful internalization of RNAi molecules into target cells. This involves crossing epithelial barriers—most notably the peritrophic matrix and midgut epithelium—and their subsequent incorporation into the cytoplasm [179], where dsRNA becomes bioavailable. Internalization can occur through multiple pathways, including clathrin-mediated endocytosis, receptor-mediated uptake, and passive diffusion.

Clathrin-mediated endocytosis is a key mechanism of dsRNA internalization in several insect species, including *S. frugiperda*, *A. pisum*, *T. castaneum*, and *D. virgifera virgifera* [180,181,182,183]. The process begins when membrane receptors recognize dsRNA, triggering the formation of clathrin-coated endocytic vesicles; essential components such as V-ATPase and Rab7 contribute to the acidification and maturation of endosomes. Within these compartments, dsRNA is released into the cytoplasm from early or late endosomes for subsequent processing into siRNAs [180,183]. Scavenger-receptor-mediated endocytosis, involving receptors such as SR-CI and Eater, has also been implicated in dsRNA uptake in insects like *Schistocerca gregaria* (Orthoptera: Acrididae) and *Drosophila melanogaster* (Diptera: Drosophilidae) [184,185]. However, inefficient endosomal escape is associated with low RNAi efficacy, as demonstrated by the accumulation of dsRNA within endosomes in lepidopteran species [183]. For instance, the low RNAi efficiency observed in *Heliothis virescens* (Lepidoptera: Noctuidae) has been attributed to the limited endosomal release of dsRNA [186].

Beyond endocytic pathways, specialized membrane proteins have also been identified as contributors to dsRNA uptake. Systemic RNA interference defective (SID) transporters and their homologs, SID-like (*SIL*) genes, encode transmembrane proteins with extracellular domains that recognize and mediate the transport of dsRNA across cellular membranes [187]. In *Caenorhabditis elegans*, SID-2 selectively recognizes and internalizes dsRNA longer than 50 base pairs from the acidic intestinal lumen via endocytosis, while discriminating against ssRNA. In contrast, SID-1 functions as a passive transmembrane channel that allows the diffusion of dsRNA into the cytoplasm, where RNAi-mediated gene silencing occurs [188]. SID-1-like genes have been identified in *S. litura*, *L. decemlineata*, *T. castaneum*, and *Nilaparvata lugens* (Hemiptera: Delphacidae), although their functionality and roles vary among species [189,190,191,192]. However, most insects lack functional SIL proteins, and SID-2 homologs have not been identified, suggesting that dsRNA uptake in these species depends on alternative pathways such as scavenger-receptor-mediated and clathrin-dependent endocytosis [60,91].

Several carriers have been explored to enhance dsRNA uptake, including functionalized carbon nanotubes (CNTs), which have demonstrated effective dsRNA delivery with low toxicity in *T. castaneum*. CNTs functionalized with polyamidoamine dendrimers improve the efficiency of gene silencing by facilitating the entry of dsRNA into the cytoplasm, resulting in a stronger phenotypic response compared to naked dsRNA [193]. Another promising strategy involves the use of CPPs, which electrostatically bind to dsRNA, promoting RNA condensation and the formation of smaller complexes [179]. CPPs such as TAT and EB1 have been evaluated for their ability to form complexes with 600 bp dsRNA, thereby enhancing endosomal escape and release into the cytoplasm [194]; additionally, nanoparticle-based encapsulation of dsRNA, as previously discussed in Section 4.1, can improve the stability of dsRNA in vivo and promote cellular uptake.

### 4.3. Intracellular dsRNA Transport and Systemic RNAi

Subsequent to its internalization into midgut epithelial cells, dsRNA is thought to be transported to the hemolymph via transcellular mechanisms. Although this process remains poorly characterized in insects, it likely involves pathways such as exocytosis, movement through channel proteins, including SID-1 homologs, and vesicle-mediated transcytosis [195,196].

In the hemolymph, dsRNA can be found either in free form or associated with proteins, forming ribonucleoprotein complexes. This interaction protects the dsRNA from nuclease degradation, enhances its stability and bioavailability, and facilitates its delivery to distant target tissues [197]. Dissemination of RNAi fragments to distal tissues enables propagation of the silencing signal. This process, known as systemic RNAi, is essential for effective gene knockdown in cells that have not been in direct contact with dsRNA [196]. Among the proteins involved, the transmembrane protein SID-1 plays a fundamental role in systemic RNAi by acting as a passive channel for the bidirectional transfer of dsRNA between cells, thereby amplifying and spreading the silencing signal throughout the organism [196,198,199]. In addition, vesicle-mediated trafficking via endocytosis also represents a significant route for dsRNA distribution, enabling movement between cellular compartments and protecting the molecule in the intracellular environment [195,200]. Furthermore, proteins such as Staufen and lipophorin have been implicated in the stabilization and delivery of dsRNA to RISC for subsequent processing [201,202].

The efficacy of the RNAi response across insect species is strongly influenced by the efficiency of intracellular dsRNA trafficking and processing. For instance, [186] reported that *L. decemlineata* exhibits higher susceptibility to RNAi than *H. virescens*, a difference that has been linked to variations in intracellular dsRNA processing. In general, Lepidoptera are considered refractory to systemic RNAi, which limits the efficacy of this approach in many species [84]. Despite this challenge, sustained gene silencing effects have been observed throughout the insect life cycle; for example, in *H. armigera*, RNAi-induced phenotypic alterations during larval stages persisted into pupal and adult stages [143].

Although the understanding of the molecular mechanisms governing dsRNA internalization and intracellular transport has improved, the pathways mediating systemic RNA interference in insects remain poorly characterized [203]. While systemic transport of RNAi signals via exosomes has been demonstrated in species such as *D. melanogaster*, *Allomyrina dichotoma* (Coleoptera: Scarabaeidae), *T. castaneum*, and *L. decemlineata* [195,204,205,206], these findings remain to be validated in other insect taxa. Similarly, nanotube-like structures have been shown to mediate intercellular transfer of dsRNA and components of the RNAi machinery in *D. melanogaster* [207], but their presence and function in other insect species remain largely unexplored. Addressing these shortcomings is essential for optimizing RNAi technologies for pest control applications.

## 5. Biological Barriers in Insects

The efficacy of RNAi-based pest control strategies in insects is influenced by their ability to overcome the multiple biological barriers that restrict dsRNA absorption and effectiveness, including both physical structures and biochemical mechanisms that promote dsRNA degradation before it can induce gene silencing. One of the main challenges is the presence of dsRNA-specific nucleases (dsRNases) in arthropod salivary secretions, hemolymph, and the digestive tract [60,208,209,210,211]. Additionally, physicochemical factors, such as gut pH, also play a significant role in the premature degradation of dsRNA, further limiting the gene silencing response [212].

### 5.1. Intestinal Digestion

Besides facilitating nutrient absorption, digestion in insects also acts as a barrier against exogenous nucleic acids, including dsRNA. Degradation may begin even during feeding through nucleases present in salivary secretions. For instance, high concentrations of dsRNase in the salivary glands of the green stink bug (*Nezara viridula*, Hemiptera: Pentatomidae) have been correlated with poor RNAi efficacy [211]. Similar degradation patterns have been reported in *A. pisum* and the tarnished plant bug (*Lygus lineolaris*, Hemiptera: Miridae) [213,214].

Once ingested, dsRNA can also be degraded due to the harsh physical and chemical environment and the enzymatic activity of the intestinal lumen. In *Diatraea saccharalis* (Lepidoptera: Crambidae), RNAi failure was linked to rapid dsRNA degradation by intestinal juice, rich in nucleases bearing endonuclease_NS and PIN domains [215]. Other examples include *L. migratoria*, where dsRNase2 is highly expressed in midgut fluid [216]. In fact, several species that are refractory to oral exposure display strong RNAi responses when the dsRNA is delivered by microinjection [67,216,217], underscoring the digestive tract as a key factor that significantly compromises RNAi efficiency.

In addition to enzymatic degradation, gut pH plays a complementary role in dsRNA instability. This factor is highly variable among insect taxa [218]. For example, lepidopteran larvae exhibit some of the most alkaline gut environments described in biological systems [219], conditions that can chemically destabilize dsRNA and enhance the activity of alkaline-dependent nucleases [220].

Interestingly, the silencing of nucleases has been reported to mitigate the detrimental effects of the insect gut environment on dsRNA stability. This approach was validated through the simultaneous silencing of *RNase5* and *RNase6* in the rice leaf folder (*Cnaphalocrocis medinalis*, Lepidoptera: Crambidae) and *RNase1* and *RNase2* in *Ceratitis capitata* (Diptera: Tephritidae) [221,222]. Furthermore, the use of nanoparticles such as layered double hydroxides, carbon quantum dots, and chitosan has shown protective effects against nuclease activity in *P. citri*, *Apolygus lucorum* (Hemiptera: Miridae), *Earias vittella* (Lepidoptera: Nolidae), and *Bombyx mori* (Lepidoptera: Bombycidae) [90,170,223,224], highlighting that such protection could be extended to additional pests and crops.

Despite the evidence, critical knowledge gaps remain. For example, it is not clear why some insect species, such as *L. decemlineata*, exhibit strong oral RNAi responses despite possessing active digestive nucleases. Although the removal of intestinal nucleases has been shown to enhance RNAi sensitivity in certain species, this approach failed in *S. gregaria*, where no improvement in RNAi efficiency was observed [225]; these findings suggest that species-specific factors, such as the localization and regulation of dsRNases or the presence of protective gut structures, may play a pivotal role in modulating RNAi efficacy.

### 5.2. Hemocoelic Barrier

Once dsRNA reaches the intestinal lumen, it must enter the hemolymph; however, to do so, it must cross the hemocoelic barrier, which prevents the spread of pathogens and foreign molecules in insects. If dsRNA fails to cross it, then it cannot be distributed systemically to other cells or tissues, limiting the effectiveness of RNAi.

The concentration and persistence of dsRNA in insect hemolymphs have been closely linked to the efficacy of RNAi. Studies conducted in *Periplaneta americana* (Blattodea: Blattidae), *Zophobas atratus* (Coleoptera: Tenebrionidae), *L. migratoria*, and *S. litura* have demonstrated that RNAi effectiveness strongly depends on the amount and stability of dsRNA circulating in the hemolymph, which in turn is influenced by the hemolymph’s nuclease activity [226]. Notably, Lepidoptera tend to exhibit the strongest enzymatic barriers to RNAi compared to Orthoptera and Coleoptera. This trend was also observed in *H. virescens*, where dsRNA is degraded within three days post-injection, whereas, in the coleopteran *L. decemlineata*, degradation takes approximately five days [186]. These observations show that the degradation and intracellular transport of dsRNA are the major factors responsible for reduced RNAi efficiency in lepidopteran insects.

Beyond species-specific differences, temporal variation in nuclease expression has also been documented. In *N. viridula*, for example, the expression of dsRNase is lower during the early nymphal stages and increases markedly at the second instar, coinciding with the onset of plant feeding [211]. These results highlight that the organism and its stage of development are factors that must be carefully considered when designing RNAi-based pest control strategies.

### 5.3. Immune Response

In pest insects, the presence of dsRNA not only triggers RNAi-mediated gene silencing but can also elicit immune responses that may impact the silencing outcome. In some insects, such as *D. melanogaster* and *Culex quinquefasciatus* (Diptera: Culicidae), dsRNA is recognized as a pathogen-associated molecular pattern (PAMP) by Dicer-2, particularly through its helicase domain. This recognition leads to the induction of immune effectors such as the Vago peptide, which subsequently activates the JAK-STAT pathway [86]. In *B. mori*, Liu et al. [227] demonstrated that dsRNA can modulate the innate immune response by altering the expression of Toll pathway genes. Furthermore, Liu et al. [228] found that the Toll-like receptor Toll9-1 was associated with the transcriptional upregulation of Dicer2, a key enzyme in the RNAi pathway. These findings suggest crosstalk between immune signaling and RNAi mechanisms. Similarly, Guan et al. [87], through transcriptome sequencing of *Ostrinia furnacalis* (Lepidoptera: Crambidae) treated with dsGFP, observed the upregulation of immune-related genes, including pattern recognition receptors typically associated with antiviral responses, and the activation of humoral immunity.

Another study reported the overexpression of apidaecins, antimicrobial peptides (AMPs) associated with humoral immune responses. The activation of endogenous antiviral mechanisms following exposure to dsRNA triggers immunostimulatory effects that result in significant alterations in the gene expression profile. Moreover, evidence suggests that the immune response induced by dsRNA is modulated by the biological context, potentially leading to a localized and specific immune activation depending on the cell type or developmental stage [118]. Flenniken and Andino [229] demonstrated that dsRNA induces a nonspecific immunostimulatory response in *A. mellifera*, characterized by a reduction in the expression of AMPs such as apidaecin, hymenoptaecin, and abaecin, along with the activation of non-canonical genes associated with antiviral immunity, including *unc-80* and *lethal(3)*. These findings suggest that the immune response to dsRNA may involve unique genes and signaling pathways distinct from classical immune routes. Therefore, immune activation and its influence on the efficacy of gene silencing must be considered carefully.

## 6. Other Factors Affecting RNAi Efficiency

Although endogenous factors significantly influence the efficiency of RNAi, external factors such as the host-associated microbiota and dsRNA resistance development may also affect the consistency and efficacy of RNAi-based gene silencing responses.

### 6.1. Environmental Interactions

One of the key advantages of dsRNA-based pest control is its rapid degradation in the environment, which contributes to a favorable biosafety profile by minimizing the risk of unintended exposure to non-target organisms. However, this same characteristic poses a significant limitation for its practical implementation. The short persistence of dsRNA in matrices such as soil, water, or plant surfaces reduces the window of biological activity, often necessitating repeated applications to maintain efficacy—thereby increasing labor and production costs.

Several studies have demonstrated that environmental degradation of dsRNA is relatively rapid. For instance, Dubelman et al. [230] reported half-lives ranging from 15 to 28 h in soils, regardless of the application method or concentration. Similarly, Joaquim et al. [231] found that *Snf7* dsRNA expressed in MON 87411 maize degraded significantly within 12 h and was undetectable after 3 days, which presents a challenge for sustained pest control. On the other hand, in aquatic environments, Fischer et al. [232] and Albright et al. [233] also reported dsRNA dissipation within three to four days, with no detectable levels after seven days. However, it remains unclear whether dsRNA partitions between the water column and sediment or binds to organic matter, potentially prolonging its persistence in specific compartments.

Other factors that play a major role in dsRNA stability include temperature, pH, UV exposure, and microbial activity. For example, although it has been reported that dsRNA is relatively stable at 50 °C, rapid degradation occurs at 60 °C, and this is accelerated by UV exposure [234,235]. In addition, although neutral pH allows dsRNA to persist for a long time, alkaline conditions accelerate degradation [236].

Microbial communities are particularly important. Fischer et al. [124] found that dsRNA degradation is primarily driven by microbial populations rather than intrinsic properties such as sequence, size, secondary structure, or biophysical properties of the matrix. This was also supported by Parker et al. [237], who emphasized the role of microbial community composition in the dsRNA dissipation. While it has been proposed that persistence results can be extrapolated to different environments [231,233], the heterogeneity of microbial populations across different matrices makes it difficult to predict dsRNA persistence in field conditions.

Currently, several attempts to enhance dsRNA stability for pest control have been reported, including nanoparticle formulations, lipid carriers, or clay nanosheets. For example, San Miguel and Scott [234] showed that foliar application of dsRNA retained biological activity for up to 28 days. Mitter et al. [172] demonstrated that dsRNA bound to layered double hydroxide (LDH) clays resisted washing and provided a controlled release for up to 30 days. Meanwhile, the trunk injection method extended dsRNA persistence to 84–141 days [162]. Additionally, lipid formulations, such as DOTAP, DOTAP + PEG, and DODMA, and other nanoparticle-based carriers that enhance efficacy, may also affect degradation kinetics, potentially increasing persistence [238]. Interestingly, it has been reported that plant roots can absorb dsRNA and remain detectable for up to 30 days [115,161]. Notably, the use of carrier compounds may be dispensable when plant cell walls are mechanically damaged [239], as such injury facilitates direct dsRNA absorption and movement within plant tissues.

Despite progress in determining dsRNA persistence in different environments—primarily agricultural—studies are still limited, which complicates the efficient and effective implementation of dsRNA on a large scale.

Paradoxically, the greater the efforts to develop strategies that enhance the persistence of dsRNA in the environment for effective and sustained pest control, the longer the exposure time of non-target organisms to dsRNA inadvertently increases, raising the risk of non-target effects. Thus, the low ecological risk of dsRNA, associated with its limited environmental persistence, may be undermined by this very strategy.

### 6.2. Interaction of the Microbiome with dsRNA

The interactions among ingested dsRNA, the host insect, and its gut microbiome have gained growing attention as key elements affecting RNAi success. Intestinal bacteria may break down dsRNA molecules, reducing the effectiveness of gene silencing; nevertheless, how dsRNA delivery influences the microbial community remains relatively underexplored.

It has been observed that certain pathogenic taxa, such as *Enterobacter aerogenes*, *Enterococcus faecalis*, and particularly *Pseudomonas putida*, can utilize the dsRNA degradation products as carbon and nitrogen sources for their growth, leading to their proliferation and causing dysbiosis. The microbial imbalance disrupts intestinal homeostasis, damages intestinal tissue, and increases permeability so bacteria can move into the hemocoel, triggering systemic infections and causing host death [240]. This suggests that the effectiveness of RNAi can be dependent on the presence of gut bacteria. This was confirmed recently by Zhang et al. [241], showing that the efficacy of dsRNA in *Plagiodera versicolora* (Coleoptera: Chrysomelidae) depended on the presence of *P. putida* and induced dysbiosis of the gut bacteria. Similarly, studies on *L. migratoria* showed that the intestinal atrophy caused by dsRNA treatment led to proliferation of opportunistic bacteria such as *E. aerogenes*, *Klebsiella pneumoniae*, and *Enterobacter asburiae* [242].

This dual role of gut bacteria—both limiting RNAi through degradation and contributing to host mortality—highlights their influence on RNAi outcomes. Although current studies modulating the intestinal microbiota to enhance dsRNA efficacy are still limited, this represents a promising avenue to design protective formulations and improve RNAi effectiveness.

### 6.3. Risks of Resistance Development

RNAi has emerged as a revolutionary third-generation pest control strategy, offering high target specificity and significantly reduced environmental impact; however, the development of resistance in target organisms represents a genuine concern in the implementation of RNAi technologies on a large scale, and it has already begun to be observed.

The first documented case of dsRNA resistance was reported by Khajuria et al. [243] in *D. virgifera virgifera*, involving transgenic maize expressing dsRNA targeting the *Snf7* gene. Subsequently, *L. decemlineata* CEAS 300 (chronically exposed adult surviving) developed >11,100-fold resistance to a dsRNA targeting the *V-ATPase subunit A* gene after nine rounds of selection with foliar application. Surprisingly, cross-resistance was observed against dsRNA targeting a different gene (*COPI β*) [244]. These findings confirm that resistance is not based on target-specific mutations but instead acts before transcript degradation and involves broader mechanisms, such as impaired uptake or intracellular processing of dsRNA [243]. Therefore, simply changing the target gene is insufficient if the underlying issue lies in dsRNA uptake. Another example of insect resistance was observed in *P. versicolora*, where the resistant population 30R showed >4110-fold resistance after seven selection cycles with dsRNA targeting the *Srp54k* gene, caused by impaired dsRNA internalization [245]. Importantly, resistance development does not necessarily entail a measurable fitness cost under laboratory conditions [246], which could facilitate the persistence and spread of resistant populations in the absence of selective pressure.

**Figure 3:**
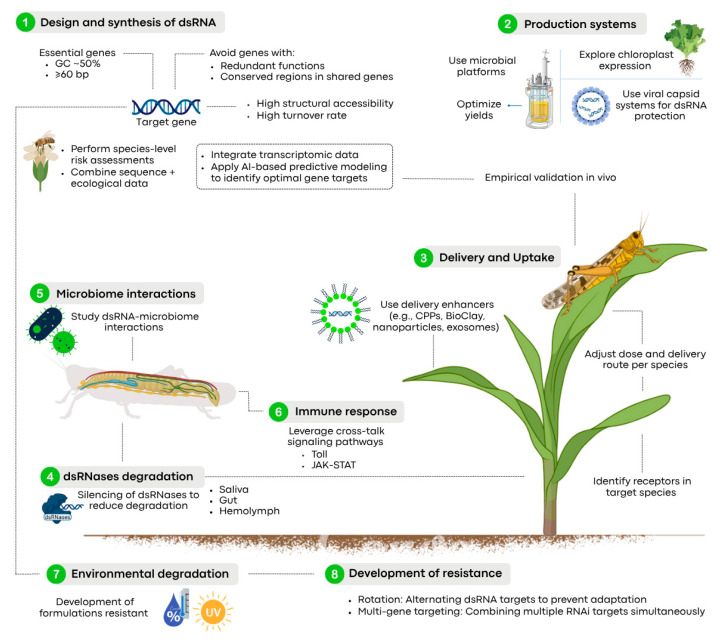
Strategies to improve the efficiency and sustainability of dsRNA-based pest control in agricultural environments.

These studies show that the development of resistance is a serious issue for the large-scale implementation of RNAi technology in agriculture. Notably, they highlight that the external application of dsRNA can rapidly promote the development of polygenic resistance. This poses a challenge for the strategy, which is often considered an alternative for bypassing the regulations that govern genetically modified plants [247,248,249,250,251]. This concern is compounded by the intrinsic variability among geographically distinct populations of the same species, which affects the consistency of RNAi efficacy, as observed in *L. decemlineata* populations [252]. To overcome dsRNA resistance, several strategies have been proposed, such as rotating different modes of action [253], implementing pyramidal approaches [254], and using formulations that contain diverse antisense oligonucleotides targeting different genes [255]. Nevertheless, further research is needed to identify the genes involved in resistance (e.g., membrane transporters or endocytosis-related proteins) in order to improve the use of RNAi in insect pest management (Figure 3).

## 7. Challenges and Opportunities

RNAi has become recognized as a highly specific strategy for pest management, distinguished by its selectivity, minimal toxicity, and environmental safety. Despite a growing number of successful studies, attempts to achieve the large-scale implementation of RNAi technology still encounter significant challenges. Primary obstacles include optimizing dsRNA design, developing efficient delivery systems, and ensuring effective cellular uptake. Furthermore, the mechanisms governing dsRNA internalization, intracellular transport, and systemic dissemination remain poorly characterized, particularly in non-model insect species. There are also ongoing concerns about the influence of RNAi on insect gut microbiota and the possible development of resistance.

A further challenge remains in attempts to achieve consistent RNAi efficacy in target pest populations while limiting off-target effects on beneficial or non-target organisms. Moreover, as RNAi-based solutions are relatively novel, regulatory frameworks governing dsRNA products, whether incorporated into genetically modified crops or applied as topical treatments, are still in their early stages. Although some regulatory progress has been made, comprehensive guidelines are necessary to properly evaluate the potential environmental and public health implications.

Despite these hurdles, several commercial dsRNA-based formulations are already available on the market, enabling their potential integration as pest control tools. As research and technological advances address current limitations, RNAi is solidifying its role as a key component in next-generation pest management, contributing to the development of more precise, sustainable, and efficient agricultural systems.

## Figures and Tables

**Table 1:** RNAi target genes evaluated in insect and mite species.

Gene	Function	mRNA Size (kb)	Degree of Conservation	Maximum Knockdown Reported	Phenotypic Effect	References
*Ion transport and calcium regulation*						
*V-ATPase*	Ion and nutrient transport; regulation of cellular homeostasis.	Subunit A, ~2.0–2.5 kb; Subunit B, ~1.5–1.8 kb; Subunit D, ~1.0–1.2 kb; Subunit E, ~0.7–0.9 kb.	Highly preserved	80%	Decreased survival and fertility of female thrips and reduced number of offspring.	[29,31,38,60,61]
*RyR*	Release of calcium (Ca^2+^) from the sarcoplasmic reticulum into the cytosol during muscle contraction.	~15.5 kb	Highly preserved	75%	Reduction in survival and emergence of adults.	[17,62]
*Hormonal and neuromuscular signaling*						
*ACE*	Hydrolysis of acetylcholine, a neurotransmitter released at the synapse to facilitate signal transmission between neurons.	~1.4–2.3 kb	Highly preserved	70%	Reduced weight gain, larval, nymphal, and adult mortality, and increased pesticide susceptibility.	[29,33,35,54]
*EcR*	Primary receptor for ecdysone, a steroid hormone that regulates the molting, metamorphosis, and reproduction processes.	~1.5–2.8 kb	Highly preserved	90%	Reduction in weight, longevity, fertility, egg laying, failure to hatch, morbidity, and mortality.	[26,37,39,50]
*Detoxification and xenobiotic metabolism*						
*CYP450*	Compounds’ bioactivation and xenobiotic metabolism.	~1.5–2.0 kb	Highly preserved	90%	Weight decreased, reduced enzymatic CPR activity, and increased pesticide susceptibility.	[12,28,32,34,63]
*Cytoskeleton, cell motility, and vesicular trafficking*						
*SNF*	Vesicular transport, formation of multivesicular vesicles, protein degradation, and cell division.	~1.2–1.5 kb	Highly preserved	94%	Larval and adult mortality.	[64,65]
*ACT*	Cell structure, movement, and division.	~1.5–2.0 kb	Highly preserved	71%	100% mortality, cessation of feeding, decreased larval weight, and altered actin filaments.	[16,46]
*TPM*	Muscle function and maintenance of cytoskeletal integrity.	~1.2–2.2 kb	Highly preserved	76%	Reduction in feeding; mortality.	[58]
*α-COP*	Vesicular transport between different compartments of the endomembrane system.	~10–15 kb	Highly preserved	82%	Mortality	[66,67]
*Energy metabolism*						
*TRE1*	Catalyzes the hydrolysis of trehalose involved in energy metabolism, chitin synthesis, and metamorphosis.	~1.9–2.9 kb	Moderately preserved	85%	Abnormal phenotypes, wing and molt deformities, alteration of genes involved in chitin biosynthesis, soft and transparent cuticle, weight loss, and mortality.	[27,68,69]
*ArgK*	Catalysis of the transfer of phosphates to arginine, participating in energy metabolism.	~0.3–1.1 kb	Highly preserved	80%	Length, weight, and pupation rate reduction, melanization, forewings and antenna malformation, and mortality.	[25,29,70,71]
*Synthesis and macromolecule processing*						
*Vg*	Provision of nutrients to oocytes and embryo development.	~5.0–5.5 kb	Moderately preserved	99%	Atrophy of oogenesis, decreased size of ovaries and eggs, low egg production, delayed oviposition periods, and lack of hatching.	[30,72]
*Chy*	Digestive proteolysis in the intestine.	~0.7–0.9 kb	Highly preserved	87%	Length and weight reduction, mortality.	[29,73]
*CHS*	Chitin biosynthesis, vital for structure, protection, and mobility.	~4.2–4.7 kb	Highly preserved	90%	Decreased chitin content, ecdysis inhibition, delayed larval growth, abnormal pupation, and mortality.	[36,59,74]

## Data Availability

No new data were created or analyzed in this study. Data sharing is not applicable to this article.
